# Child Labor and Environmental Health: Government Obligations and Human Rights

**DOI:** 10.1155/2012/938306

**Published:** 2012-12-18

**Authors:** Joseph J. Amon, Jane Buchanan, Jane Cohen, Juliane Kippenberg

**Affiliations:** ^1^Human Rights Watch, 350 Fifth Avenve, 34th Floor, New York City, NY 10118, USA; ^2^Human Rights Watch, Neue Promenade 5, 10178 Berlin, Germany

## Abstract

The Convention concerning the Prohibition and Immediate Action for the Elimination of the Worst Forms of Child Labour was adopted by the International Labour Organization in 1999. 174 countries around the world have signed or ratified the convention, which requires countries to adopt laws and implement programs to prohibit and eliminate child labor that poses harms to health or safety. Nonetheless, child labor continues to be common in the agriculture and mining sectors, where safety and environmental hazards pose significant risks. Drawing upon recent human rights investigations of child labor in tobacco farming in Kazakhstan and gold mining in Mali, the role of international human rights mechanisms, advocacy with government and private sector officials, and media attention in reducing harmful environmental exposures of child workers is discussed. Human rights-based advocacy in both cases was important to raise attention and help ensure that children are protected from harm.

## 1. Introduction

The Convention concerning the Prohibition and Immediate Action for the Elimination of the Worst Forms of Child Labour, adopted by the International Labour Organization (ILO) in 1999 and ratified by 174 countries, prohibits hazardous labor for children under the age of 18. Prohibited work includes work with dangerous machinery or in dangerous locations, work that exposes children to physical, psychological, or sexual abuse, and work with hazardous substances, agents, or processes [1, 2].

Despite the widespread acceptance of this prohibition, the International Labour Organization (ILO) has estimated that 115 million children aged 5–17 years engage in hazardous work annually [3]. Much of this work occurs in developing countries with poor regulatory oversight, where children have limited access to health care or basic information on health risks and preventive measures.

Children working in mining and agriculture sectors face especially high risks to their health and well-being. More children work in the agricultural sector than any other sector, and exposure to fertilizers, pesticides, and herbicides is common [3]. Specific agricultural crops, such as tobacco, which is grown in 130 countries worldwide [4], pose direct health risks to children through exposure to nicotine from tobacco leaves [5]. Between 10 and 15 million artisanal and small scale gold miners, including 4.5 million women and 300,000 children [6], face exposure to toxic metals, especially mercury, which is used to amalgamate gold in more than 70 countries [7].

This paper presents two case studies of environmental health hazards and child labor: in tobacco farming in Kazakhstan and in artisanal gold mining in Mali. Human rights-based advocacy in both cases, including advocacy with government authorities and private companies, engagement of human rights treaty mechanisms, and media exposure of abuses were important tools to raise attention to hazardous environmental exposures of child workers and to help ensure that children are protected from harm.

## 2. Methods

The case studies presented here stem from two distinct human rights investigations in Kazakhstan and Mali. Each used mixed methods, including in-depth interviews; analysis of international human rights law, national legislation, and government health, labor, and environmental policies; and review of relevant health and environmental studies. More detailed methods are presented elsewhere [8, 9].

### 2.1. Kazakhstan

Field research in Kazakhstan took place between March and September 2009 and December 2009 and February 2010. Interviews were conducted with 68 migrant tobacco workers from 39 families, ages from 19 to 50, and five child migrant tobacco workers under age 18. The majority of interviews took place in the Enbekshikazakh district of Almaty province. Representatives from UN agencies (the International Labour Organization (ILO), the International Labor Organization's International Programme on the Elimination of Child Labour (ILO-IPEC), the International Organization for Migration (IOM), United Nations Development Fund for Women (UNIFEM)), labor unions (the International Union of Food, Agricultural, Hotel, Restaurant, Catering, Tobacco and Allied Workers' Associations (IUF)), and international and Kazakhstani NGOs were also interviewed. Officials from Kazakhstan's Ministry of Internal Affairs, the Ministry of Labor and Social Protection, and the Ombudsman Office met with researchers, as did representatives from Philip Morris International (PMI) and Philip Morris Kazakhstan (PMK). Additional in-depth interviews with 27 adult migrant workers employed in tobacco farming in the Enbekshikazakh district of Almaty province took place in July 2010 and September 2011to assess changes in labor practices including child labor and occupational health protections following the release of primary findings and advocacy meetings.

### 2.2. Mali

Field research in Mali was carried out between February and April 2011 in Bamako and in mining areas in Western and Southern Mali. Interviews were conducted in three mining sites in Kéniéba circle, in the Kayes region of Western Mali; in Worognan (Mena commune) in Kolondiéba circle; and in the Sikasso region of Southern Mali. Over 150 people, including 41 children working in mining areas (24 boys and 17 girls), were interviewed. Interviews were conducted with parents and guardians of child workers, adult miners, teachers and principals, health workers and health experts, village chiefs, *tombolomas *(traditional mining chiefs), NGO activists, and representatives of UN agencies and donor governments. The Minister of Labor and Civil Service and his staff, as well as officials in the Ministry of Mines, the Ministry of Health, the Ministry of Environment and Sanitation, the Ministry of Education, and the Ministry of Promotion of Women, Child and Family Affairs of the Malian government were interviewed, as were local government officials in Kéniéba and Kolondiéba circles.

### 2.3. Human Subject Protections

All research was undertaken with informed consent of participants. Interviews were conducted in a private setting, and anonymity was offered to individuals discussing their personal experiences. Interviews with family members of child workers took between 45 and 90 minutes. Interviews with children were typically shorter, between 15 and 30 minutes. In Mali, interviews were conducted in French or a local language with the assistance of a translator. In Kazakhstan, interviews were conducted in Kyrgyz or Uzbek with the assistance of a translator translating into Russian. In a few instances in Kazakhstan interviews were conducted in Russian. In both settings, researchers used a semistructured questionnaire. In Mali, questions focused upon the access to education and health care, health status and labor conditions. In Kazakhstan, questions focused on labor conditions, including evidence of forced labor and induced indebtedness, as well as payments and wages; access to education; use of hazardous substances; and passport withholding and freedom of movement.

Pseudonyms are used in presenting the testimony below. The investigation was funded by Human Rights Watch, who does not generally identify its work as “research,” defined as seeking to develop “generalizable knowledge” [10]. Rather, its investigations aim to examine laws and policies, document and respond to specific human rights issues, monitor human rights conditions, and assess human rights protections. Each of these purposes is consistent with what has been defined as “public health non-research” (such as surveillance, monitoring, and evaluation) [11] or practice [10]. Because public health non-research and practice also raise ethical and human participant protection issues, all investigations conducted by Human Rights Watch staff are subject to rigorous internal review, and external ethics and subject-area experts are consulted when investigations involve particularly difficult settings, populations, or issues [12].

## 3. Case Studies

### 3.1. Kazakhstan

Kazakhstan is the ninth-largest migrant-receiving country in the world [13], with significant numbers of migrant workers, including children, engaged in tobacco farming. An exact estimate of the number of children working in tobacco is challenging, however, due to the absence of monitoring and reporting mechanisms. In 2006 the ILO estimated that children constituted up to 60 percent of the workforce in tobacco farming [14].

Tobacco farming in Kazakhstan entails a range of agricultural tasks performed by both adults and children including growing tobacco plant seedlings, transplanting seedlings to fields, watering, weeding, fertilizing, and applying pesticides, then harvesting the leaves by hand, stringing and hanging the leaves for curing, steaming the leaves to prepare them for packing, and packing them in bales.

Tobacco cultivation is painstaking manual work and poses significant health risks for children, including exposure to pesticides and green tobacco sickness (GTS), which is caused by the absorption of nicotine through the skin from contact with tobacco leaves [15]. Protective clothing can decrease the magnitude of GTS, as can delaying work in wet fields until tobacco leaves are dry [16].

Despite a prohibition on child labor in tobacco farming in Kazakhstan, in-depth interviews with migrant workers found that child labor in tobacco farming was common and had been going on for years. Children performed some, or all, of the same labor-intensive work as adults. For example, Alym A., from Karatash, Kyrgyzstan, said that his 14-year-old daughter “planted tobacco seedlings, watered the tobacco plants, hoed, picked the leaves, and strung, dried, steamed and pressed the leaves.” Child workers typically worked long hours: “The children work like we do, doing everything. Typically we work from 7 a.m. to 7 or 8 p.m.” stated Sabir S., who was interviewed working with his son and daughter, aged 15 and 13 years.

Children and migrant farm workers often had little information on the consequences of the exposure to tobacco or pesticides, common symptoms of exposure, or knowledge of protective measures. Sharapat Sh., who worked with her adult son and 15-year-old daughter, said “We do not know of any harm.” Most workers interviewed lacked protective clothing to use during the tobacco harvest. Although some had gloves, many workers, including children, did not: “We do not have special protective clothing,” stated Alym A. Umut U., said that her four children, aged 10, 11, 13, and 14, strung tobacco, grew seedlings, planted, and applied fertilizer and pesticides, but had no protective clothes.

The child labor practices identified violated numerous provisions in Kazakhstani law. For example, a Ministry of Labor and Social Protection order regarding hazardous professions explicitly prohibits the employment of children under 18 in tobacco [17], and the Labor Code of Kazakhstan prohibits employment of persons under age 18 in harmful or hazardous working conditions [18]. Following the investigation, the government was encouraged to address this gap between law and practice through increased inspections and sanctions against violators. Advocacy also focused on the government's failure to ensure access to schools for migrant children, in violation of its commitments under the UN Convention on the Rights of the Child [19], the International Covenant on Economic, Social, and Cultural Rights (ICESCR) [20], and the ILO's Worst Forms of Labor Convention [21].

Evidence of child labor and other abuses in tobacco farming in Kazakhstan was also presented to PMI and its subsidiary, PMK, which is the sole purchaser of tobacco in Kazakhstan. PMI, along with other global tobacco companies, has had policies on child labor for more than a decade [22] but have been criticized for not addressing structural causes of child labor and not having effective monitoring strategies [23]. In Kazakhstan, PMI and PMK were regarded as key actors responsible for, and capable of, bringing labor conditions in line with international standards, including by eliminating hazardous child labor. Specific recommendations were made to strengthen measures to prevent child labor and support alternatives to work for children of migrant workers.

Following numerous meetings, PMI and PMK agreed to undertake programs that aim to eliminate hazardous child labor in Kazakhstan, including by monitoring for child labor through regular and unannounced inspections; conducting additional trainings with farmers and parents regarding the harmful effects of child labor; advocating with the Kazakhstani authorities regarding access to schools for migrant children; and sponsoring summer camps, a community center, vocational training, and other activities for children as alternatives to working. In 2010 PMI also launched a new global Agricultural Labor Policy, which elaborates on its previous policies prohibiting hazardous child labor and develops guidelines and requirements for farmers, growers, and suppliers in the PMI supply chain regarding child labor and other human rights abuses. As part of this policy, PMI is undertaking training and monitoring in the 30 countries from which it sources tobacco [24].

Interviews with migrant tobacco workers in the Enbekshikazakh region in 2010 and 2011 indicated that PMI and PMK had taken certain steps to address human rights problems, including hazardous child labor, alternatives to work for children, and access to public schools for undocumented migrant children. At the same time, researchers again witnessed children working, and some interviewees indicated that their children or other migrant workers' children continued to work in tobacco farming.

Evidence of abuses against migrant child workers was also presented to the UN Committee on Economic, Social and Cultural Rights, ahead of its May 2010 periodic review of Kazakhstan's compliance with the ICESCR [25]. In its concluding observations, the committee raised issues concerning migrant workers and their children, stating that it “is deeply concerned at the precarious situation of migrant workers who are employed without contracts in tobacco plantations and are, together with their families, vulnerable to exploitation and abuse,” and calling on the government to conduct its own evaluation “with a view to establishing mechanisms that enforce the relevant Labour Code provisions” [26]. In addition, information on abuses was given to the UN Special Rapporteur on Contemporary Forms of Slavery in advance of her visit to Kazakhstan in July 2012. With respect to child labor specifically, in a press statement following the visit, the Special Rapporteur expressed concerns about legal obstacles that limit the ability of the children of undocumented migrant workers to attend school and have access to health care [27].

Complementing direct advocacy with the government, PMI, PMK, United Nations agencies, and other actors, was a strategy of engagement with local and international media. Research results were publically released in Almaty at a press conference, and the findings were widely covered in the Kazakhstan and international media. A video including interviews with migrant workers, expert testimony, photographs, and video footage was also produced and made available on websites, including YouTube, where to date the video has received over 17,000 views [28].

### 3.2. Mali

The west African country of Mali is among the poorest in the world, with a population of 14.5 million people, one-half of whom live below the international poverty line of US$1.25 day. Between 20,000 and 40,000 children are estimated to work in Mali's artisanal gold mines [9]. Most children work in mining alongside a parent or sibling. Others are sent to live and work with another family or live and work by themselves.

Children in artisanal gold mining often perform dangerous, backbreaking work, including digging shafts, transporting and crushing ore. Children also mix mercury with crushed ore to bind gold and create an amalgam, which is then heated to evaporate the mercury, leaving gold behind. Exposure to mercury through contact or inhalation can cause developmental problems and neurological symptoms including tremors, twitching, vision impairment, headaches, and memory and concentration loss [29]. Higher levels of mercury exposure may result in kidney failure, respiratory failure, and death. Mercury exposure can also affect men's and women's reproductive health, reducing fertility and causing miscarriages [30, 31]. Mercury is particularly harmful to fetuses and infants and can be transmitted in utero and through breast milk [31]. In addition, even if they are not working, small children inhale the mercury vapor when they are present near amalgamation sites.

Of 33 children interviewed working in artisanal mining, 14 said they carried out amalgamation using mercury. The youngest was six years old. Susanne D., 11, told us how she works with mercury: “Once the ore is panned, you put a bit of mercury in. You rub the ore and the mercury with your two hands. Then, when the mercury has attracted the gold, you put it on a metal box and burn it. When I have finished, I sell the gold to a trader. I do this daily. I usually get about 500 CFA francs (equivalent of US$ 1.08) for the gold.”

Several girls mentioned back pains, headaches, and general fatigue caused by gold panning. Aminata C., a 13-year-old girl from Baroya mine, told us: “I do gold panning and mixing (amalgamation). I often feel pain everywhere, I have headaches and stomach aches.” While some children knew that mercury was dangerous, many had never heard of any health risk associated with the use of mercury, and none knew why mercury was dangerous or how to protect themselves from mercury exposure.

Fatimata N., a 15-year-old girl from Burkina Faso, said: “I put the mercury in with the sand and the water. I mix it with my bare hands. Then I put the mercury in my *pagne* (a wrap-around skirt). The mercury that I squeeze out, I keep it in a small plastic bag. I also burn it. I have never heard that this is unhealthy.” Mohamed S., 16, said: “No one has ever told me that mercury is dangerous. We are told that it has magic powers… to capture the gold out of minerals.”

Although Malian government regulations list mercury as a dangerous product [9], miners said that gold traders regularly deal in mercury and provide it to children. Malian law prohibits hazardous labor for anyone under age 18 [32], and a national list of types of hazardous work specifically prohibits the use of child labor in almost all activities relating to artisanal gold mining.

In direct talks with the Malian government, and in public statements following the investigation, the government was urged to implement current labor laws. The government was also encouraged to develop a national strategy on mercury reduction that would seek to end mercury use by children; expand training for healthcare workers and improve access to testing and treatment for children suffering from mercury poisoning. Recognizing that Mali's government will be unable to take action without international support, international donors were urged to provide financial and technical assistance for education in mining areas, targeting efforts towards the withdrawal of children from artisanal gold mining and to address mercury exposure.

In response, the government agreed to consider the particular situation of children in its formulation of a national action plan on mercury in artisanal gold mining and invited expert stakeholders to provide input. It has also mentioned the issue of mercury use by children in its recent national strategy on toxic chemicals. At the same time, however, the Ministry of Mining made public statements attempting to minimize the existence of child labor in Mali's mines. Local and international media covered the issue widely and prompted heated debate when the head of the Malian Mining Chamber denied the existence of child labor in artisanal gold mining on Radio France Internationale. In the US, NBC produced a 16-minute documentary on child labor in Mali's gold mines that was watched by over 3.4 million people [33].

International donors and UN agencies responded to the results of the investigation by integrating mercury, and child Labor issues more strongly in their work. For example, the ILO has agreed to address the threat of mercury in its ongoing work on child labor in Mali's mines, and the United Nations Industrial Development Organization (UNIDO) has sought input on child labor issues in a new project on mercury reduction and social conditions in artisanal gold mining in West Africa. The 2012 US Department of Labor's report on child labor judged that Mali had made a “moderate advancement in efforts to eliminate the worst forms of child labor” during the year, citing, among other measures, the participation in the ILO program. Nonetheless, the report concluded that “mechanisms to fight child labor remain inefficient and some laws are not harmonized, leaving children unprotected from exploitative child labor” [34].

Finally, evidence of child labor was made available to several international companies trading in gold, and recommendations were designed for gold traders. A boycott on the purchase of artisanal gold was not recommended, because it was believed that it would be detrimental to the fragile economy in artisanal gold mining areas. Nonetheless, one Dubai-based company decided to suspend its gold trade with Mali. Large-scale mining companies active in Mali were also approached for support to address mercury exposure and child labor in artisanal settings, with limited results.

## 4. Discussion

The two case studies we highlight here represent a small fraction of the environmental health hazards faced by child laborers daily around the world. In addition to tobacco farming and gold mining, children perform hazardous work in other types of agriculture and mining, as well as in fishing, domestic labor, manufacturing, and other economic sectors. In the silver mines of Bolivia, approximately 120,000 children perform backbreaking work and risk their lives hauling dynamite [35]. In India, two million children work in hazardous sectors such as brick making, firework manufacturing, and quarrying [36]. Children as young as eight work on sugarcane plantations in El Salvador [37] and in toxic tanneries in Bangladesh [38].

Since 2010, more than 400 children have died of lead poisoning in a region of artisanal gold mining in Northern Nigeria, and despite promises by the Nigerian government to implement safe-mining practices and clean up contaminated sites, little action has been taken by the government and thousands of children remain at risk [39]. In addition to Kazakhstan and Mali, Human Rights Watch has documented hazardous child labor in numerous countries, including El Salvador [37], Bangladesh [38], the United States [40], Morocco [41], India [42], Guinea [43], Senegal [44], Indonesia [45], Malaysia [46], and Uzbekistan [47].

In Kazakhstan and Mali, human rights-based research, including qualitative in-depth interviews and legal and policy analysis, was used to engage a diverse set of actors—including UN agencies, international donors, government officials, business, and media—to comprehensively address hazardous child labor and seek sustainable solutions. As both case studies demonstrate, even countries that have domestic laws prohibiting hazardous child labor may have gaps in enforcement and may need to be challenged to implement effective enforcement policies. Advocacy at the national level is complemented by the attention at the international level, where efforts to end hazardous child labor have increased in the last decade, particularly from United Nations agencies.

Reducing harms from child labor in tobacco farming and exposure to mercury have both been the focus of international efforts for more than a decade. A resolution from a February 2003 ILO tripartite meeting on the future of employment in the tobacco sector called for the ILO Director General to continue to promote the Minimum Age Convention and the Worst Forms of Child Labor Convention and to assist in their application specifically in the tobacco sector. The resolution also called on all parties engaged in implementing these conventions to adopt “concrete measures to eliminate child labor in the tobacco chain” [48]. In 2005, the ILO started a global campaign called “Minors out of Mining!” to combat child labor. It brought together governments, trade unions, and employers from around the world and aimed to eliminate child labor in mining by 2015 [49]. However, these initiatives have stalled with little concrete achievement or followup. ILO has complained about a lack of commitment among some of the signing parties and a lack of funding for work on child labor in mining. Seven years later, the initiative is largely forgotten.

In the past few years, the United Nations Environment Program has begun a global effort to negotiate a legally binding treaty to reduce the use of mercury. Initial negotiations in 2011 and 2012, however, had little attention to child labor or the right to health. Using the ILO convention and CRC as baseline guarantees of rights protections, NGOs have advocated for specific language addressing these issues, and Latin American and African governments—including Mali, which represents the African region (with Nigeria)—have begun championing their inclusion in the treaty. Although the process is ongoing, the current draft of the treaty now obligates governments to take measures to protect children and women of childbearing age from the effects of mercury use in artisanal gold mining. However, Western governments have sought to block efforts by Latin American countries to further expand attention to health in the treaty.

The World Health Organization's Framework Convention on Tobacco Control (FCTC) provides another example of a missed opportunity to address environmental health hazards of child workers. The FCTC has been ratified by 176 countries and requires signatories to take steps to reduce the health consequences of tobacco. Specific measures obligate countries to enact laws that control the tobacco industry's production and promotion of tobacco and promote education about the dangers of tobacco use and secondhand smoke [50]. Yet, despite these progressive measures and requirements for annual reporting of progress against a range of indicators [51], no explicit attention is given in the treaty, or reporting instrument, to child labor. Although each country is asked to report on the number of workers in tobacco growing, disaggregated by full-time, part-time, and seasonal workers and by gender, no request is made to estimate child tobacco workers. While Article 18 of the treaty entitled “Protection of the environment and the health of persons” does specifically address the health of tobacco farmers, it merely asks governments to have “due regard” for their protection, and no specific indicators are included in the annual reporting requirements related to the availability of protective clothing for workers, promotion of information on health risks of tobacco leaf or pesticide application, or estimates of the incidence of GTS. A July 2012 paper by the FCTC working group on Articles 17 and 18 presents a set of guidelines related to child labor [52], but a rights-based approach to tobacco control, founded upon more explicit country monitoring and linkage to other human rights treaty monitoring, is needed [53].

The UN High Commissioner for Human Rights has also begun work on environmental health and human rights. In March 2012, the Human Rights Council appointed an independent expert on the issue of human rights obligations related to the enjoyment of a safe, clean, healthy, and sustainable environment. This expert will engage governments, business, and civil society, among others, to address human rights abuses related to environmental degradation. This role could serve as a focal point for environmental health issues related to child labor where, like in Mali and Kazakhstan, a complex set of factors require a multisectorial approach and advocacy strategy.

Finally, in both Mali and Kazakhstan outreach to the media has been an important advocacy tool, putting pressure on government and private sector actors to respond to documented abuses. Multinational companies, attuned to the potential negative impact on their brands, are increasingly sensitive to allegations of poor environmental and labor practices. In Kazakhstan, media coverage helped increase pressure on the government and PMI and PMK. In Mali, where artisanal gold production involves a complex international supply chain, media coverage focused more on the role of the government, prompting both new efforts to address child labor and a defensive reaction from the Malian Ministry of Mining and Mining Chamber. It also generated interest in new fair trade gold standards and raised attention to the issue of child labor and mercury poisoning among international donors.

## 5. Conclusions

In both Kazakhstan and Mali, human rights advocacy has been effective in drawing attention to the environmental health risks of child laborers and in engaging government, business, civil society, and UN actors in seeking evidence-based and sustainable solutions. Advocacy contributed to greater awareness among stakeholders and to specific changes in policy and practice in the countries examined, as well as globally. However, many challenges remain, and the elimination of hazardous child labor requires ongoing, sustained advocacy, government commitment and, often, international donor support. The lack of integration of issues related to child labor, environmental protection, and health at the national and international levels, in different government ministries, international treaties, and UN and donor funding programs and initiatives, frequently poses a barrier to effective and immediate action.

Environmental health risks associated with child labor can only be ended if different actors—governments, civil society, UN, donors, and companies—prioritize its elimination, give their full political support to it, and provide financial support to efforts aimed at ending it. Linking scientists, health care providers, and human rights advocates together can provide for new alliances, strategies, and opportunities to document the prevalence and consequences of hazardous child labor and to eliminate it.
